# Survey of *Strongyloides papillosus* Infection in Goats and Sheep in Part of China Using RAA-LFD Assay

**DOI:** 10.1155/tbed/5004834

**Published:** 2025-10-25

**Authors:** Yongde Xu, Wanting Li, Muhammad Tahir Aleem, Yuheng Zhang, Jilata Amu, Mingmin Lu, Xiaokai Song, Lixin Xu, Ruofeng Yan

**Affiliations:** ^1^MOE Joint International Research Laboratory of Animal Health and Food Safety, College of Veterinary Medicine, Nanjing Agricultural University, Nanjing 210095, China; ^2^Center for Gene Regulation in Health and Disease, Department of Biological, Geological, and Environmental Sciences, College of Sciences and Health Professions, Clevaland State University, Clevaland 44115, Ohio, USA

**Keywords:** epidemiological investigation, goat and sheep, RAA-LFD, rapid detection, *Strongyloides papillosus*

## Abstract

Strongyloidiasis caused by *Strongyloides papillosus* is a significant parasitic disease affecting the health and productivity of small ruminants globally. In this study, a novel recombinase-aided amplification (RAA) combined with lateral flow dipstick (LFD) assay was developed and validated for the rapid and specific detection of *S. papillosus* infection in goats and sheep, targeting the 18S ribosomal RNA (18S rRNA) gene. A total of 815 fecal samples were collected from nine provinces in China, encompassing major goats and sheep production regions. The RAA-LFD assay exhibited high sensitivity, with a minimum detection limit of 15 eggs per gram (EPG) of feces. Clinical fecal examination yielded a positive rate of 62.30% (433/695), while the RAA-LFD assay achieved a positive rate of 58.28% (475/815), indicating that the diagnostic accuracy of the RAA-LFD assay is consistent with that of fecal examination. Compared with conventional fecal examination, the RAA-LFD assay offers superior rapidity, portability, and sensitivity, presenting a valuable diagnostic tool for large scale epidemiological surveillance and point-of-care applications, particularly in resource constrained environments.

## 1. Introduction

With the rapid intensification of global animal husbandry, the prevention and control of parasitic diseases have become critical for improving animal health, enhancing production efficiency, and ensuring the safety and quality of animal derived food products [1, 2]. Parasitic infections adversely affect growth, reproduction, and immune competence in livestock, increasing susceptibility to secondary infections and contributing to significant economic losses. *Strongyloides papillosus*, a globally distributed and highly pathogenic intestinal nematode, poses a major threat to ruminants such as cattle, sheep, and goats [3–6]. The complex life cycle and dual transmission routes (cutaneous and oral) of *S. papillosus*, coupled with its significant pathogenicity manifested as wasting, diarrhea, anemia, and growth retardation that highlight its detrimental impact on livestock productivity and the urgent need for comprehensive control strategies [7, 8]. In young animals, *S. papillosus* infection can impair growth and development, weaken immunity, and increase susceptibility to other pathogens, leading to higher morbidity and mortality rates [9]. High stocking density and confined housing conditions in intensive farming systems facilitate the rapid transmission of *S. papillosus* [10], resulting in significant economic losses due to reduced productivity and increased veterinary costs.

Conventional diagnosis of *S. papillosus* predominantly depends on parasitological methods, with fecal egg detection serving as the principal approach [11, 12]. This technique relies on morphological identification and quantification of parasite eggs in fecal samples; however, its sensitivity is limited, particularly in low-intensity infections or during the prepatent period, where sporadic and minimal egg shedding may result in false-negative outcomes and delayed diagnosis [13]. Diagnostic accuracy is influenced by factors such as sample collection procedures, processing techniques, and examiner expertise, potentially leading to false-positive or false-negative results; conventional methods like larval culture and morphological identification are labor-intensive, time-consuming, and require specialized skills and laboratory facilities [13]. These limitations render conventional diagnostic approaches insufficient to meet the demands of modern livestock production, which necessitate rapid, accurate, and high-throughput solutions for large-scale herd screening and field-level disease surveillance. Recent advancements in molecular biology have transformed the diagnostics of parasitic diseases, enabling highly sensitive, specific, and rapid detection methods [14–17]. Among these, recombinase-aided amplification (RAA) technology combined with lateral flow dipstick (LFD) detection has demonstrated unique advantages and broad application prospects [18–21]. RAA, a novel isothermal nucleic acid amplification technique, has emerged as a robust diagnostic tool owing to its simplicity, rapidity, and accuracy [22, 23]. The RAA amplification system utilizes recombinase, single-stranded DNA-binding protein (SSB), and DNA polymerase, wherein recombinase-primer complexes facilitate homologous sequence recognition and strand invasion, stabilized by SSB, enabling polymerase-driven DNA synthesis [24]. The RAA reaction facilitates rapid and efficient gene amplification at a constant temperature (37–42°C) within 20–30 min, and when coupled with LFD detection using a labeled probe, enables visual identification of amplicons without the requirement for specialized instrumentation [25].

In recent years, RAA-LFD assays have been successfully applied to the detection of multiple human and animal pathogens [26, 27]. In this study, a rapid and visually interpretable RAA-LFD assay with high specificity and sensitivity was developed for the detection of *S. papillosus*, and its diagnostic performance was systematically validated under field conditions for potential application in clinical diagnostics, epidemiological surveillance, and outbreak investigations.

## 2. Materials and Methods

### 2.1. Parasite, Bacterias, and Clinical Samples

**Figure 1:**
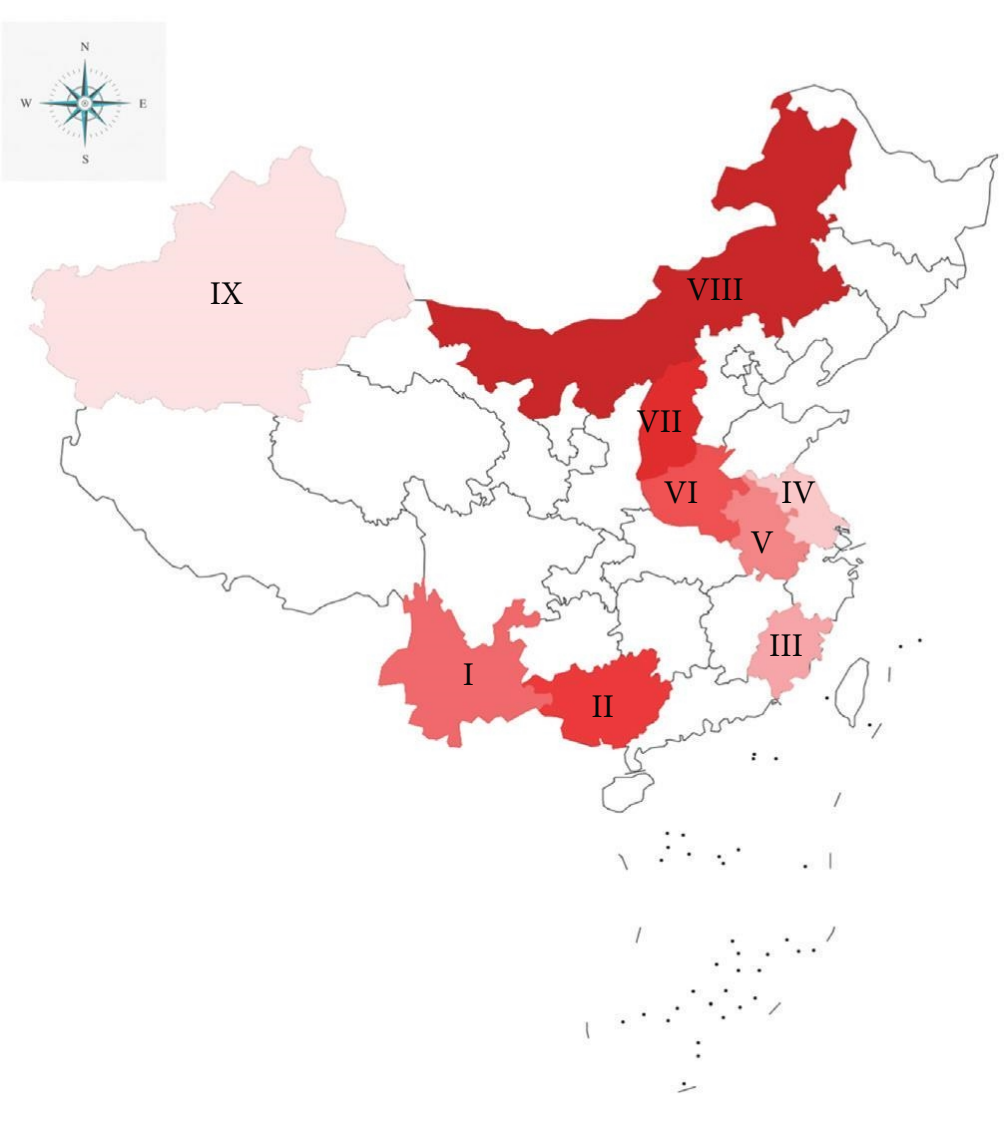
Geographic distribution of fecal samples. A total of 815 fecal samples were collected, including (Ⅰ) 90 goat samples from Zhaotong City, Yunnan Province; (Ⅱ) 90 goat samples from Baise City, Guangxi Zhuang Autonomous Region; (Ⅲ) 90 goat samples from Quanzhou City, Fujian Province; (Ⅳ) 111 goat samples from Nantong City, Jiangsu Province; (Ⅴ) 90 goat samples from Chuzhou City, Anhui Province; (Ⅵ) 90 goat samples from Luoyang City, Henan Province; (Ⅶ) 90 goat samples from Jinzhong City, Shanxi Province; (Ⅷ) 94 sheep samples from Tongliao City; Nei Mongol Autonomous Region; (Ⅸ) 70 sheep samples from Urumqi City, Xinjiang Uygur Autonomous Region.

*S. papillosus*, *Haemonchus contortus*, *Eimeria christenseni*, *Escherichia coli*, *Salmonella typhimurium*, *Toxoplasma gondii*, and *Trichinella spiralis* were stored at our laboratory. A total of 815 fecal samples were collected from nine geographical regions in China (Figure 1) from September to October 2024, and kept at 4°C. Two to three farms were sampled in each region, with each farm housing a population of approximately 200–300 sheep or goats. In the Xinjiang Uyghur Autonomous Region, herders had administered levamisole for deworming 1 month prior to sampling.

### 2.2. Extraction of DNA

The genomic DNA of *S. papillosus*, *H. contortus*, *E. christenseni*, *E. coli*, *S. typhimurium*, *T. gondii*, and *T. spiralis* were extracted following the instructions of the SteadyPure Universal Genomic DNA Extraction Kit (Accurate Biotechnology [Hunan] Co., Ltd., China). All the DNA samples were stored at −20°C.

### 2.3. Cloning of *S. papillosus* 18S ribosomal RNA (18S rRNA) Gene

The specific primers (Sp-F and Sp-R; Table 1) were designed based on the coding sequence of the *S. papillosus* 18S rRNA gene (GenBank: KX138391.1). The PCR reaction was prepared in a total volume of 25 μL, containing 1 μL of each primer (10 μM), 12.5 μL of 2×PrimeSTAR Max Premix (Takara, Shanghai, China), 2 μL of DNA template, and 8.5 μL of nuclease-free water. The cyclic terms were used accordingly: the first denaturation for 5 min at 98°C (1 cycle), denaturing (30 s at 98°C), annealing (30 s at 55°C), extension (10 s at 72°C) (30 cycles), and final extension (72°C for 5 min [1 cycle]). The PCR products were purified using the FastPure Gel DNA Extraction Mini Kit (Vazyme, Nanjing, China), followed by cloning into pUC57 vector. The recombinant plasmid identified as positive was sent to Qingke Biotechnology Co., Ltd. (Beijing, China) for gene sequencing.

### 2.4. Optimization the Method for DNA Extraction From Feces

For Method 1, DNA extraction was modified from the method described by Zou et al. [28]. Briefly, 1 g of positive fecal sample from goat was homogenized with 800 μL of SDS lysis buffer (10 mM Tris-HCl [pH 8.0], 2 M NaCl, 7 mM SDS, and 8 mM EDTA) and 20 μL of proteinase K (10 mg/mL), followed by incubation at 55°C for 8 min. A diameter of 5 mm Whatman Number 1 filter paper disc was immersed in the lysate for 2 min to allow DNA binding. The disc was then transferred to 600 μL of wash buffer (10 mM Tris-HCl [pH 8.0], 0.1% Tween-20) and gently agitated for 2 min. Finally, the disc was placed in 40 μL of elution buffer (10 mM Tris-HCl [pH 7.5]) for 1 min to elute the bound DNA. For Method 2, DNA extraction was performed in parallel using the Stool DNA Kit (Omega, Nanjing, China).

To evaluate the sensitivity of Method 1, eggs were counted and aspirated under a microscope at quantities of 1, 5, 10, 15, 20,25, 30, and 35, then added to 1 g of negative goat feces to prepare artificial positive fecal samples. About 1 g of negative goat feces served as the negative control. PCR amplification was carried out on the extracted DNA, incorporating the plasmid containing 18S rRNA gene as the positive control. The reaction and thermal cycling conditions were identical to those detailed in Section 2.3., using primers RAA-F and RAA-R (Table 1).

### 2.5. Establishment of RAA-LFD Assay

#### 2.5.1. Design of Primers and Probes

Primers and probes were designed as per the requirements of the RAA nucleic acid amplification kit (test strip method) (Kelan Biotechnology Co., Ltd., Nanjing, China). The downstream primer was labeled with biotin. The probe was modified with a FAM fluorophore at its 5′end. There was a single-base gap at a position 30 bp from the 5′end, which was modified with THF residue, and the 3′end was blocked by phosphorylation (Table 1). Primers and probes were synthesized and labeled by Qingke Biotechnology Co., Ltd. (Beijing, China).

The reaction system (25 μL) was prepared with buffer V, 12 μL; purified water, 7 μL; SpRAA-F, 1 μL; SpRAA-R, 1 μL; SpRAA-P, 1 μL; DNA template, 1 μL; magnesium acetate, 2 μL. The reaction tubes were briefly centrifuged and incubated in a 37°C water bath for 30 min. Following amplification, the RAA products were diluted 10-fold with PBST, and 50 μL of each dilution was applied to the sample pad of the LFD for visual analysis. After 1 min of reaction at room temperature, results were interpreted as positive if both the test (T) and control (C) lines appeared simultaneously, negative if only the C line was visible, and invalid if any other pattern was observed.

#### 2.5.2. Optimization of Probe Concentrations and Reaction Times

Probe concentrations were optimized at 1000, 100, 10, and 1 nM, with all reactions incubated for 30 min. Incubation times were systematically evaluated at 5, 10, 15, 20, and 25 min using a thermal cycler set at 37°C.

#### 2.5.3. Sensitivity Test

Under the abovementioned optimized conditions, the sensitivity of the RAA-LFD assay was determined using 10-fold serially diluted standards of a recombinant plasmid harboring the 18S rRNA gene. The initial plasmid concentration of 9.37 × 10^9^ was diluted to 9.37 × 10^4^ copies/μL. To assess the detection limit in biological matrices, artificially fecal samples were prepared by spiking 1 g of parasite-free sheep feces with 1, 5, 10, 15, 20, 25, 30, and 35 eggs added. Genomic DNA extracted from these spiked samples was then subjected to RAA-LFD detection.

#### 2.5.4. Specificity Test

Under the abovementioned optimized conditions, the DNA of *S. papillosus*, *H. contortus*, *E. christenseni*, *E. coli*, *S. typhimurium*, *T. gondii*, and *T. spiralis* were used as the templates for the RAA-LFD assay, and nuclease-free water was instead of the template as a negative control.

#### 2.5.5. Repeatability Test

Genomic DNA was extracted from previously confirmed positive stool samples and subjected to three independent RAA-LFD assays. A negative control was included in each experimental replicate to validate assay reproducibility.

### 2.6. Detection of Clinical Samples Using RAA-LFD Assay

All samples were analyzed by RAA-LFD assay. To evaluate clinical diagnostic performance, parallel testing was conducted on 695 samples using fecal examination: (1) fecal flotation and (2) larval culture. For flotation-based egg detection, 2 g of fresh feces were homogenized in 58 mL saturated saline, filtered through a 40-mesh sieve, and aliquoted into test tubes filled to form a meniscus. Coverslips were placed on the tube mouths for 15 min and then transferred to slides for microscopic examination. For larval culture, 10 g of feces was molded into a hemispherical shape in a flat dish, moistened if necessary, covered, and incubated at 28°C for 6 h. Larvae were subsequently harvested from the fecal juice, mounted on slides, and examined microscopically.

### 2.7. Statistical Analysis

In this study, statistical analysis was performed using GraphPad Prism 6.0 (GraphPad Software Inc., San Diego, CA, USA). Associations between potential risk factors and *S. papillosus* infection were evaluated using chi-square or Fisher's exact tests, supplemented by Monte Carlo simulation. A *p*-value <0.05 was considered statistically significant.

## 3. Results

### 3.1. Amplification of the 18S rRNA Gene

**Figure 2:**
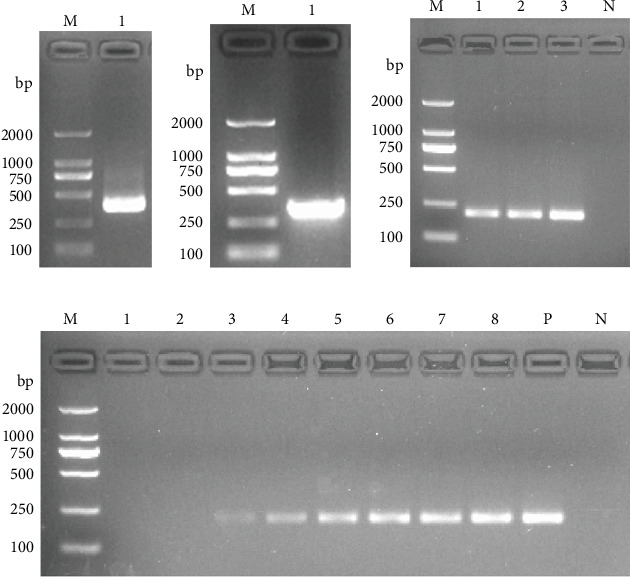
*S. papillosus* 18S rRNA gene amplification by PCR. Lane M: DNA marker DL2000. (A) Amplification of 18S rRNA gene (362 bp) using primers Sp-F and Sp-R. Lane 1: genomic DNA. (B) Amplification of 18S rRNA gene (362 bp) using primers Sp-F and Sp-R. Lane 1: positive plasmid containing 18S rRNA gene. (C) Amplification of 18S rRNA gene (240 bp) using primers RAA-F and RAA-R. Lane 1: genomic DNA extracted by Method 1; lane 2: genomic DNA extracted by Method 2; lane 3: positive plasmid containing 18S rRNA gene; lane N: negative control. (D) Sensitivity analysis of Method 1, a 240 bp product was amplified using primers RAA-F and RAA-R. Lanes 1–8: genomic DNA extracted from 1 g of goat feces spiked with 1, 5, 10, 15, 20, 25, 30 and 35 eggs, respectively; lane P: positive plasmid containing 18S rRNA gene, lane N: negative control.

PCR amplification targeting the genomic DNA of *S. papillosus* was performed using specific primers, yielding a target fragment of approximately 362 bp (Figure 2A). Similarly, PCR amplification of the recombinant positive plasmids using the same primer set generated an amplicon of the expected size (Figure 2B).

### 3.2. Evaluation of DNA Extraction Efficiency and Assay Sensitivity

The parasite-positive fecal samples were used to evaluate and compare the viability of two DNA extraction methods (Method 1 and Method 2). Agarose gel electrophoresis confirmed successful DNA extraction from both methods, as indicated by distinct bands corresponding to the target amplicons (Figure 2C). However, Method 1 was selected for subsequent experiments due to its operational simplicity and shorter processing time.

The sensitivity of Method 1 was evaluated using fecal samples prepared by spiking 1 g of negative sheep feces with 1, 5, 10, 15, 20, 25, 30, and 35 eggs, respectively. Following PCR amplification of DNA extracted by Method 1, no amplification products were detected in samples containing 1–5 eggs per gram (EPG). Faint bands were observed in samples with 10 EPG, whereas clear and consistent bands appeared in samples with 15 or more EPG, with band intensity increasing proportionally to the number of eggs in the feces (Figure 2D). These findings indicate that the limit of detection (LoD) of Method 1 is 15 EPG of feces.

### 3.3. Optimization of RAA-LFD Detection Conditions

#### 3.3.1. Optimization of Probe Concentrations and Reaction Times

**Figure 3:**
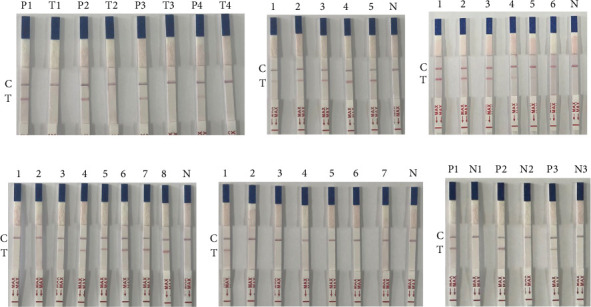
Development and optimization of RAA-LFD assay. (A) Optimization of probe concentrations. Test strips P (positive plasmid) and T (negative control) with probe concentrations of 1000 nM (1), 100 nM (2), 10 nM (3), and 1 nM (4). (B) Optimization of reaction times. Test strips 1–5: RAA reaction times of 5, 10, 15, 20, and 25 min, respectively; test strip N: negative control. (C) Sensitivity analysis using 18S rRNA positive plasmid. Test strips 1–6: plasmid concentrations of 9.37 × 10^9^, 9.37 × 10^8^, 9.37 × 10^7^, 9.37 × 10^6^, 9.37 × 10^5^, and 9.37 × 10^4^ copies/μL, respectively; test strip N: negative control. (D) Sensitivity analysis using *S. papillosus* DNA from artificial positive feces. Test strips 1–8: DNA extracted from 1 g feces of goats spiked with 1, 5, 10, 15, 20, 25, 30 and 35 eggs, respectively; test strip N: negative control. (E) Specificity test. Test strips 1–7: genomic DNA of *S. papillosus*, *H. contortus*, *E. christenseni*, *E. coli*, *S. typhimurium*, *T. gondii*, and *T. spiralis*, respectively; test strip N: negative control. (F) Repeatability test. Test strips P1–P3: positive samples for three independent tests; test strips N1–N3: negative controls for three independent tests.

The RAA-LFD method was optimized by testing probe concentrations of 1000, 100, 10, and 1 nM. Positive plasmid controls (Group P) were compared with experimental groups lacking DNA templates (Group T). Distinct T-lines were observed at probe concentrations of 1000, 100, and 10 nM, while faint T-lines were detected at 1 nM. Based on these results, 10 nM was selected as the optimal probe concentration for subsequent analyses (Figure 3A).

Reaction time optimization revealed that bands became progressively brighter over time. Although faint bands appeared after 5 min, clear and stable bands were observed at 10 min. Therefore, a reaction time of 10 min was chosen for all further experiments (Figure 3B).

#### 3.3.2. Sensitivity Test

The plasmid was serially diluted 10-fold to generate concentrations ranging from 9.37 × 10^9^ to 9.37 × 10^4^ copies/μL for the assay, and results indicated that the lowest detectable limit (LDL) of the RAA-LFD assay at the plasmid level was 9.37 × 10^5^ copies/μL (Figure 3C). Additionally, evaluation of the assay using DNA extracted from fecal samples with varying egg counts demonstrated a minimum detection capability of 15 EPG of feces (Figure 3D).

#### 3.3.3. Specificity Test

The nucleic acid of *S. papillosus* and other pathogens were used as the templates for the specific detection of the RAA-LFD assay. Results showed that DNA from *S. papillosus* produced a test band on the dipstick, while DNA from other pathogens showed negative results (Figure 3E), indicating that the RAA-LFD assay for *S. papillosus* detection has good specificity with no cross-reaction with other pathogens.

#### 3.3.4. Repeatability Test

Egg DNA extracted from positive sheep fecal samples was subjected to three independent RAA-LFD assays, with a negative control included in each replicate. The results showed consistent outcomes across the three independent experiments, indicating that the assay exhibits good reproducibility (Figure 3F).

### 3.4. Survey of *S. papillosus* Infection in China

A total of 815 clinical samples were analyzed for *S. papillosus* infection. The overall positivity rate was 58.28% (Table 2), with significant age-related differences: the 0–1 year age group exhibited a 70.77% prevalence (385/544) versus 33.21% in the ≥1 year age group (90/271) (*p* < 0.0001). After controlling for regional variables, animals aged 0–1 year demonstrated a 4.87-fold increased infection risk (OR = 4.870; 95% CI: 3.566–6.660). Species analysis revealed a marginally higher prevalence in goats (58.98%, 384/651) compared to sheep (55.48%, 91/164), though this difference was not statistically significant (*p*=0.426; OR = 1.154; 95% CI: 0.816–1.620). Geographical analysis uncovered pronounced prevalence disparities (Table 2). Northern regions exhibited the highest detection rates: Nei Mongol Autonomous Region (86.17%; OR = 37.38; 95% CI: 15.23–83.05; *p* < 0.0001), Shanxi Province (75.56%; OR = 18.55; 95% CI: 7.921–42.00; *p* < 0.0001), and Henan Province (70.00%; OR = 14.00; 95% CI: 6.174–30.69; *p* < 0.0001). Southern provinces showed intermediate prevalence, ranging from Fujian (46.67%; OR = 5.250; 95% CI: 2.395–11.12; *p* < 0.0001) to Guangxi Zhuang Autonomous Region (72.22%; OR = 15.60; 95% CI: 6.804–34.57; *p* < 0.0001). Yunnan Province (63.33%; OR = 10.00; 95% CI: 4.676–22.23; *p* < 0.0001) exhibited environmentally influenced high prevalence. Eastern coastal regions demonstrated lower rates, such as Jiangsu Province (34.23%; OR = 3.123; 95% CI: 1.457–6.501; *p*=0.0032). The Xinjiang Uygur Autonomous Region (14.29%) served as the reference category, showing a significantly reduced risk compared to all other regions(all *p* ≤ 0.0032).

Regional analysis revealed distinct geographical disparities in infection patterns (Table 2). Notably, four provinces exceeded the 60% prevalence threshold: Yunnan Province (63.33%), Guangxi Zhuang Autonomous Region (72.22%), Shanxi Province (75.56%), and Nei Mongol Autonomous Region (86.17%). These findings underscore the necessity of implementing the RAA-LFD assay in region-specific control programs to address the observed spatial heterogeneity of *S. papillosus* infections.

**Figure 4:**
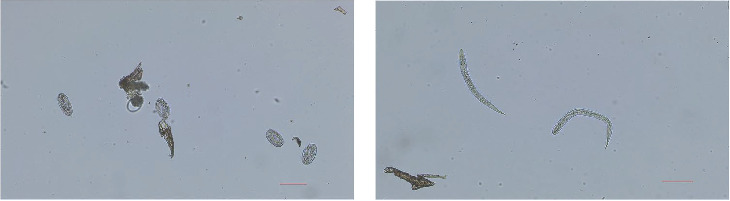
Morphology of *S. papillosus* in clinical feces. (A) Eggs and (B) first-stage larvae. Scale bar: 100 μm.

Microscopic examination based on the morphological identification of *S. papillosus* eggs (Figure 4A) and first-stage larvae (Figure 4B) demonstrated a positivity rate of 62.30%. The RAA-LFD assay achieved a 58.28% positivity rate, showing diagnostic accuracy comparable to that of conventional microscopy. These findings confirm the field applicability of RAA-LFD, with robust diagnostic performance and operational practicality under heterogeneous environmental conditions.

## 4. Discussion

Strongyloidiasis, caused by the parasitic nematode *S. papillosus*, primarily affects ruminants—with goats, sheep, and cattle as key hosts—colonizing their digestive tract. During larval migration, the pathogen can damage the skin, lungs, and small intestine, posing a significant threat to livestock industry development [29]. *S. papillosus* exhibits a remarkably wide geographical distribution, with particularly high prevalence rates in both tropical and temperate livestock production systems; contemporary epidemiological surveillance across endemic regions of Asia, Africa, and Europe reveals substantial variations in infection rates, suggesting potential associations with climatic, husbandry, or host genetic factors [5, 6, 30–32]. Currently, no effective commercial vaccines are available for prevention or treatment. Accordingly, developing a simple, rapid, and visual diagnostic tool is critical for timely diagnosis, disease management, and molecular epidemiological investigations.

In this study, the RAA-LFD assay showed a detection limit of 15 EPG in fecal samples and 9.37 × 10^5^ copies/μL for the target gene, indicating a promising application in clinical trials. However, there is a gap in this assay compared with the detection limit of 1 copy/μL achieved in SARS-CoV-2 [25] and *Plasmodium* [33] tests, which may be attributed to its longer reaction time. In our study, 10 min was used in RAA, while that it was 30 or 15 min for SARS-CoV-2 and *Plasmodium* tests. Another reason may be the DNA extraction method. Fecal-derived DNA extraction inherently impairs amplification efficiency due to nucleic acid degradation, inhibitors, and contaminations. The discrepancy between the detection limit of 15 EPG and 9.37 × 10^5^ copies of the target gene likely stems from the fact that the eggs of *S. papillosus* used in this study were hatching, and the copies of rDNA might be more than those in normal cells. Similar reports have been found in *Xenopus laevis*, where the copies of rDNA increase about 1000-fold during oocytes development [34].

The striking sensitivity of RAA-LFD in those studies also highlights the substantial room for optimization in our current system. Future work should prioritize refining primer and probe sequences to enhance specificity and binding affinity, particularly for regions of the target DNA that are conserved across parasitic strains. Collectively, these findings highlight RAA-LFD as a powerful tool for early and accurate parasite detection. While this rapid test aids in preventing outbreaks, comprehensive management remains essential. Epidemiological patterns of parasitic infections are strongly influenced by goat age and species. Younger animals exhibit heightened susceptibility, attributable to immature immune systems, whereas adult individuals develop partial resistance [9]. Similarly, species-specific differences are evident: local breeds, through long-term adaptive evolution, display enhanced resistance to endemic parasites, in contrast to introduced species, which face elevated infection risks [7, 10, 35]. Integrating RAA-LFD with rapid testing thus enables real-time, large-scale screening in resource-limited settings, enhancing the precision of epidemiological surveillance and targeted interventions.

The global expansion of livestock production has elevated the risk of parasitic infections. The present study revealed widespread *S. papillosus* infection in China, characterized by geographical heterogeneity and age-dependent susceptibility, which is consistent with previous studies [36–38]. This disparity likely stems from: (1) immature adaptive immunity in juveniles, which delays Th2-mediated responses [39]; (2) inadequate passive immunity transfer [40]; and (3) underdeveloped gut microbiota. Age-related grazing behavior and weaning stress further elevate exposure to infective larvae [41, 42]. These findings highlight the need for age-specific interventions during the immunologically vulnerable pre-weaning period [43]. A control framework is proposed, combining timed anthelmintic treatment (6–8 weeks postpartum), nutritional support, and pasture management.

No significant difference in *S. papillosus* prevalence was observed between goats (58.98%) and sheep (55.48%), indicating limited host specificity under field conditions. This challenges historical species-dependent susceptibility reports and suggests: (1) similar grazing ecology homogenizes exposure risks, (2) comparable immunological responses across ruminants [44], and (3) local parasite adaptation to host genotypes [45]. The 3.5% prevalence difference falls within diagnostic variability thresholds, implicating extrinsic factors (husbandry/environment conditions) as primary transmission drivers. These findings support unified control strategies across species, with species-specific pharmacokinetic considerations for anthelmintics [46].

The geographical variation in the prevalence of streptococcal infections across China was significant, ranging from 14.29% in the Xinjiang Uygur Autonomous Region to 86.17% in the Inner Mongolia Autonomous Region, highlighting the critical role of environmental factors. The low positivity rate in the Xinjiang Uygur Autonomous Region may be attributed to deworming with levamisole administered by local herders 1 month prior to sampling. The highest prevalence was observed in temperate grasslands (Nei Mongol Autonomous Region, 86.17%; Shanxi Province, 75.56%), where moderate summer temperatures (20–25°C) and concentrated rainfall create optimal conditions for larval development and pasture contamination [47]. Conversely, Xinjiang's arid climate (14.29% prevalence; annual precipitation <150 mm, evaporation rate >1500 mm) limits larval survival through desiccation. Subtropical provinces (including Guangxi Zhuang Autonomous Region, 72.22%; Yunnan Province, 63.33%) show intermediate-to-high prevalence, likely sustained by humid conditions and warm temperatures that enable year-round transmission, though UV exposure and forage diversity may mitigate transmission intensity. Coastal regions with intensive farming (Jiangsu Province, 34.23%; Fujian Province, 46.67%) exhibit lower rates, potentially reflecting biosecurity measures and anthelmintic usage in managed systems. These findings underscore the necessity for region-specific control strategies tailored to local climatic and ecological drivers of transmission.

## 5. Conclusion

This study revealed a notable prevalence of *S. papillosus* in goats and sheep across China and demonstrated that the RAA-LFD assay is a rapid, sensitive, and reliable diagnostic tool, showing strong agreement with conventional fecal examination methods.

## Figures and Tables

**Table 1:** Primers and probe.

Methods	Primers and probe	Sequences (5′–3′)
PCR	Sp-F	AAAGATTAAGCCATGCATGTG
	Sp-R	GCCTGCTGCCTTCCTTGGATGT

PCR	RAA-F	AACCGCGGAAAGCTCATTATAACAGCTA
	RAA-R	GTCACAACCATGGTAGGCCAATACCCTA

RAA-LFD	SpRAA-F	AACCGCGGAAAGCTCATTATAACAGCTA
	SpRAA-R	Bio-GTCACAACCATGGTAGGCCAATACCCTA
	SpRAA-P	FAM-GGATAACTGAGGTAATTCTTGAGCTAATAC[THF]CGCTATTTATACCAC-C3

**Table 2:** Positive rate and risk factors for *S. papillosus* infection tested by RAA-LFD and fecal examination.

Variable	RAA-LFD				Fecal examination			
	Samples	% (Positive number)	Odds ratio (95% CI)	*p*–Value	Samples	% (Positive number)	Odds ratio (95% CI)	*p*–Value
Age								
0–1 year	544	70.77 (385)	4.870 (3.566–6.660)	<0.0001*⁣*^*∗∗∗∗*^	424	77.12 (327)	5.248 (3.741–7.262)	＜0.0001*⁣*^*∗∗∗∗*^
≥1 year	271	33.21 (90)	1	—	271	39.11 (106)	1	—
Species								
Goat	651	58.98 (384)	1.154 (0.8156–1.619)	0.4263	531	63.46 (337)	1.230 (0.08615–1.766)	0.2693
Sheep	164	55.48 (91)	1	—	164	58.53 (96)	1	—
Location								
Yunnan Province	90	63.33 (57)	10 (4.676–22.23)	<0.0001*⁣*^*∗∗∗∗*^	90	70.00 (63)	10.23 (4.885–21.40)	<0.0001*⁣*^*∗∗∗∗*^
Guangxi Zhuang Autonomous Region	90	72.22 (65)	15.6 (6.804–34.57)	<0.0001*⁣*^*∗∗∗∗*^	60	76.67 (46)	14.41 (6.158–32.86)	<0.0001*⁣*^*∗∗∗∗*^
Fujian Province	90	46.67 (42)	5.250 (2.395–11.12)	<0.0001*⁣*^*∗∗∗∗*^	90	54.44 (49)	5.240 (2.564–10.52)	<0.0001*⁣*^*∗∗∗∗*^
Jiangsu Province	111	34.23 (38)	3.123 (1.457–6.501)	0.0032*⁣*^*∗∗*^	111	39.63 (44)	2.879 (1.399–5.660)	0.0031*⁣*^*∗∗*^
Anhui Province	90	56.67 (51)	7.846 (3.580–16.66)	<0.0001*⁣*^*∗∗∗∗*^	60	63.33 (38)	7.573 (3.445–17.34)	<0.0001*⁣*^*∗∗∗∗*^
Henan Province	90	70 (63)	14.00 (6.174–30.69)	<0.0001*⁣*^*∗∗∗∗*^	60	80.00 (48)	17.54 (7.214–42.80)	<0.0001*⁣*^*∗∗∗∗*^
Shanxi Province	90	75.56 (68)	18.55 (7.921–42.00)	<0.0001*⁣*^*∗∗∗∗*^	60	81.67 (49)	19.53 (7.842–45.19)	<0.0001*⁣*^*∗∗∗∗*^
Nei Mongol Autonomous Region	94	86.17 (81)	37.38 (15.23–83.05)	<0.0001*⁣*^*∗∗∗∗*^	94	88.30 (83)	33.08 (13.85–79.34)	<0.0001*⁣*^*∗∗∗∗*^
Xinjiang Uygur Autonomous Region	70	14.29 (10)	1	—	70	18.57 (13)	1	—
Total	815	58.28 (475)	—	—	695	62.30 (433)	—	—

*Note:* “1” represents the reference group. *⁣*^*∗∗*^(*p* < 0.01) and *⁣*^*∗∗∗∗*^(*p* < 0.0001) indicate statistical significance. The low positive rate in Xinjiang Uygur Autonomous Region was due to recent deworming.

## Data Availability

The data that support the findings of this study are available from the corresponding author upon reasonable request.
